# Supplementary material for: DnaJC7 specifically regulates tau seeding
Source: bioRxiv. 2023 Mar 16:2023.03.16.532880. Preprint. [Version 1] doi: 10.1101/2023.03.16.532880 (PMC10055123; doi:10.1101/2023.03.16.532880)
Supplement: Supplement 1 [file NIHPP2023.03.16.532880v1-supplement-1.pdf]

bioRxiv preprint doi: https://doi.org/10.1101/2023.03.16.532880; this version posted March 16, 2023. The copyright holder for this preprint (which was not certified by peer review) is the author/funder, who has granted bioRxiv a license to display the preprint in perpetuity. It is made available under aCC-BY 4.0 International license.

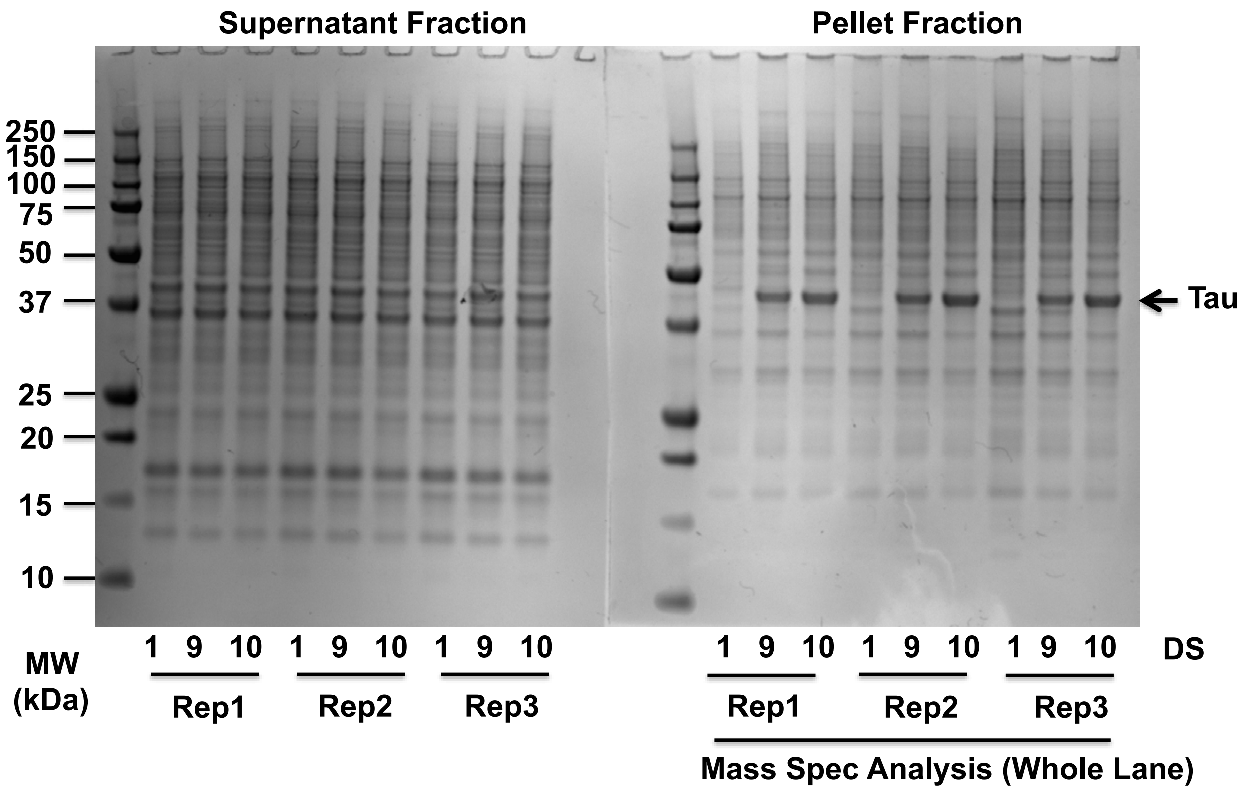

Figure 1 – Supplement 1. Partial purification of tau aggresomes.
Sarkosyl-soluble and sarkosyl-insoluble fractions were run on gels and proteins were stained with SimplyBlue protein stain. DS10 (contains tau aggregates) but not DS1 (lacks tau aggregates) insoluble fractions featured a significant enrichment of tau RD-YFP. Whole lanes for pellet fractions were analyzed by mass spectrometry (biological triplicates). The DS9 strain cell line is included as a tau-aggregate containing control. Source data for this figure are provided in Figure 1 - Supplement 1 - Source Data 1.

bioRxiv preprint doi: https://doi.org/10.1101/2023.03.16.532880; this version posted March 16, 2023. The copyright holder for this preprint (which was not certified by peer review) is the author/funder, who has granted bioRxiv a license to display the preprint in perpetuity. It is made available under aCC-BY 4.0 International license.

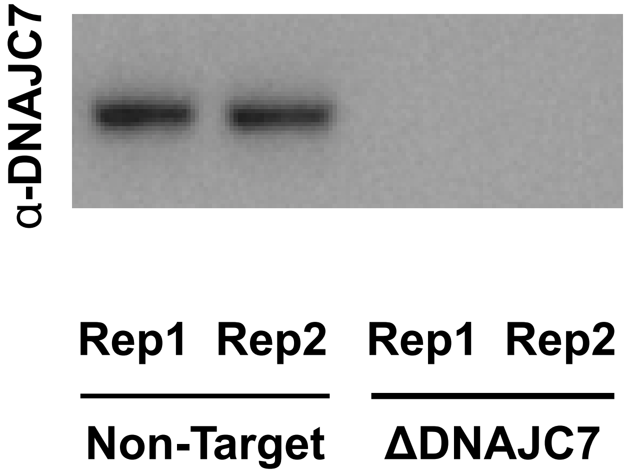

Figure 2 – Supplement 1. Western blot confirms DnaJC7 KO in (OFF1::DS10) cells.
Immunoblotting for DnaJC7 confirms that DnaJC7 is knocked out in the Tet-regulated tauRD-YFP aggregate line (OFF1::DS10). Source data for this figure are provided in Figure 2 - Supplement 1 - Source Data 1.

bioRxiv preprint doi: https://doi.org/10.1101/2023.03.16.532880; this version posted March 16, 2023. The copyright holder for this preprint (which was not certified by peer review) is the author/funder, who has granted bioRxiv a license to display the preprint in perpetuity. It is made available under aCC-BY 4.0 International license.

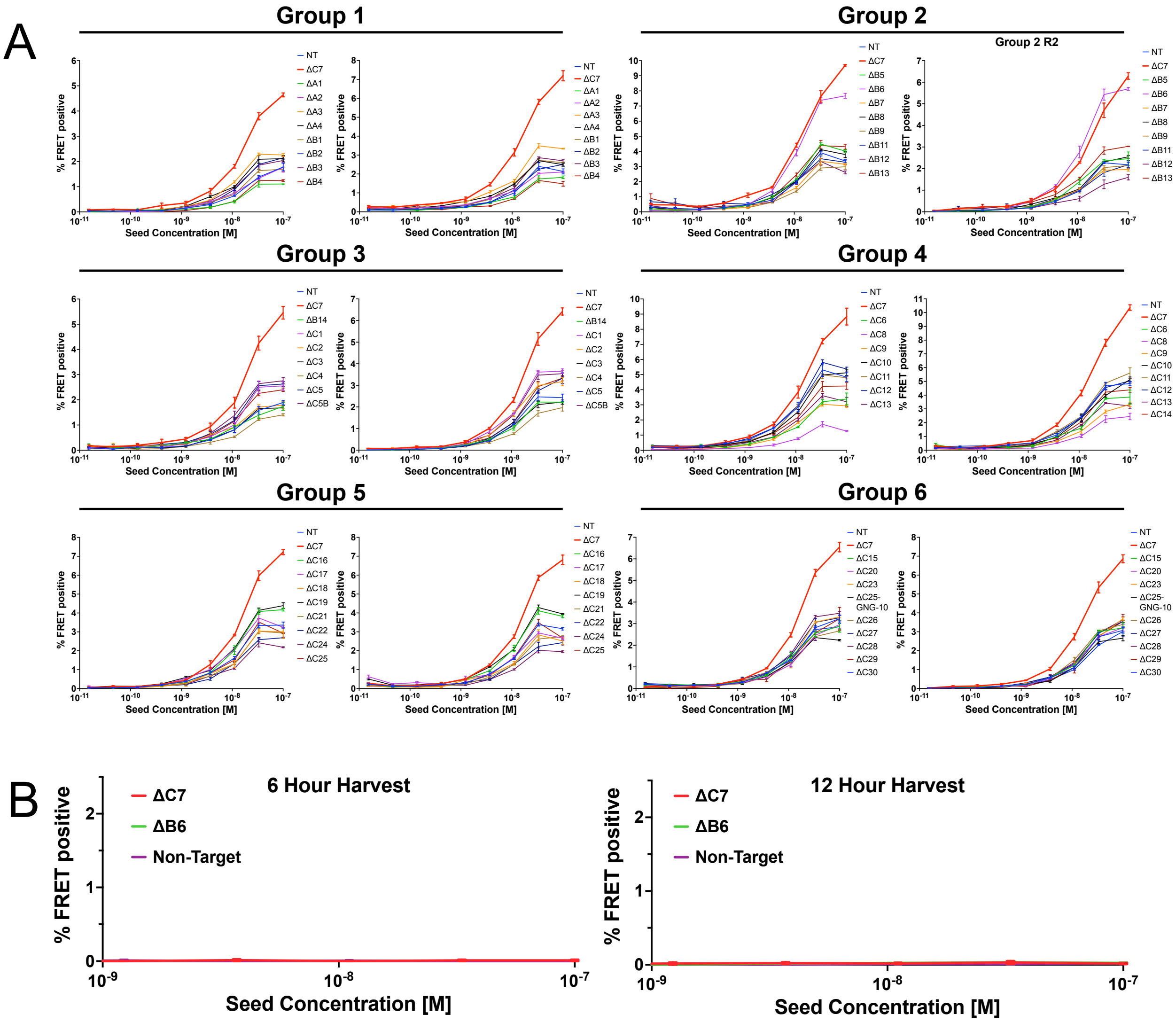

Figure 3 – Supplement 1. All individual groups of the JDP CRISPR screen.
A) Full tau dose titrations for all batches of individual knockouts of JDPs on tau seeding in biosensor cells, quantified as FRET signal via flow cytometry. Cells were seeded with a dose titration of sonicated tau fibrils. Knockout of DnaJC7 (ΔC7, red) and the nontargeting control (NT, blue) are highlighted in each batch. **B)** Extended time course harvesting of the tau seeding assay for DnaJC7 KO (ΔC7, orange), DnaJB6 KO (ΔB6, green), and nontargeting control (Non-Target, purple) cells harvested at 6 h and 12 h timepoints. All error bars represent SEM of three technical replicates.

bioRxiv preprint doi: https://doi.org/10.1101/2023.03.16.532880; this version posted March 16, 2023. The copyright holder for this preprint (which was not certified by peer review) is the author/funder, who has granted bioRxiv a license to display the preprint in perpetuity. It is made available under aCC-BY 4.0 International license.

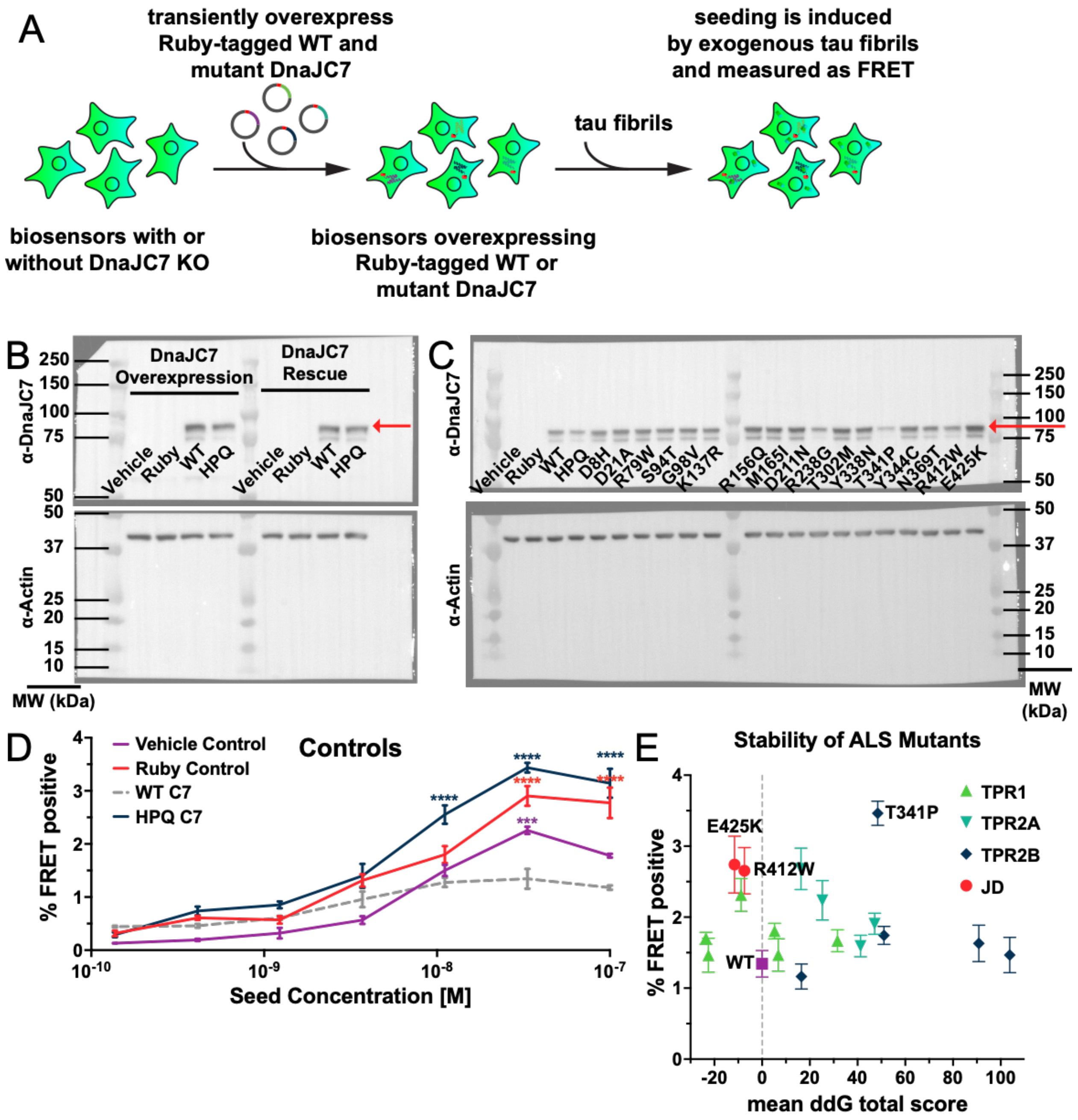

Figure 5 – Supplement 1. Stability and expression of DnaJC7 mutants in tau biosensors.

A) Schematic showing tau biosensor cells with or without DnaJC7 KO transiently overexpressing different Ruby-tagged gRNA-resistant DnaJC7 constructs. Cells are allowed to express the constructs for two days before being plated for the seeding assay. **B)** Immunoblotting for DnaJC7 confirms expression of the Ruby-WT DnaJC7 and Ruby-DnaJC7 (HPQ) mutant constructs in tau biosensor cells without (Overexpression) and with endogenous DnaJC7 knocked out (Rescue). Ruby fusion constructs are highlighted by a red arrow. **C)** Immunoblotting for DnaJC7 confirms expression of the Ruby-WT DnaJC7 and Ruby-DnaJC7 ALS mutant constructs in tau biosensor cells with endogenous DnaJC7 knocked out. Ruby fusion constructs are highlighted by a red

bioRxiv preprint doi: https://doi.org/10.1101/2023.03.16.532880; this version posted March 16, 2023. The copyright holder for this preprint (which was not certified by peer review) is the author/funder, who has granted bioRxiv a license to display the preprint in perpetuity. It is made available under aCC-BY 4.0 International license.

arrow. **D)** Positive and negative controls utilized in the rescue of DnaJC7 KO in tau biosensor cells with ALS-associated mutants of DnaJC7, colored as follows: Vehicle control, purple; Ruby control, orange; WT DnaJC7, grey dashed; HPQ mutant, dark blue. Error bars represent SEM of three technical replicates. **E)** Rosetta-calculated mean Gibbs free energy shift (ddG) of the ALS-associated mutants of DnaJC7 vs their rescue seeding with 33 nM of tau fibrils. Grey dashed line denotes a mean ddG total score of 0. Mutants are colored according to their domain localization: TPR1, green; TPR2A, teal; TPR2B, dark blue; JD, orange. Error bars represent SEM of three technical seeding replicates. * = $p<0.05$, ** = $p<0.01$, *** = $p<0.001$, **** = $p<0.0001$. Source data for this figure are provided in Figure 5 - Supplement 1 - Source Data 1.
